# Field Surface Type and Season-Ending Lower Extremity Injury in NFL Players

**DOI:** 10.1155/2024/6832213

**Published:** 2024-11-08

**Authors:** William F. McCormick, Mitchell J. Lomis, Matthew T. Yeager, Nicholas J. Tsavaris, Christopher D. Rogers

**Affiliations:** ^1^University of Georgia, Athens, Georgia, USA; ^2^College of Medicine, FIU Herbert Wertheim College of Medicine, Miami, Florida, USA; ^3^Department of Orthopaedic Surgery, Medical College of Georgia at Augusta University, Augusta, Georgia, USA

**Keywords:** field, football, injury, lower-extremity, NFL, surface

## Abstract

There is growing concern over the safety of artificial turf when it comes to the incidence of player injuries. The artificial surfaces can withstand more play, are cheaper to maintain, and are more predictable. However, there is concern that this beneficial durability comes at the expense of the forgiveness of the surface, leading to more injuries. In this study, we aim to compare the incidence of in-game season-ending lower extremity injuries on natural and artificial playing surfaces in the National Football League (NFL) during the 2020, 2021, and 2022 seasons. For this study, we used publicly available data to determine and classify the specific injury, where the injury occurred, field surface type, and duration of recovery. All data were collected, and significance was determined using two-sample *T*-tests. Only in-game injuries were included in this study. Over the course of 2020, 2021, and 2022, there were 199 season-ending lower-extremity injuries. Of these, 79 occurred on natural turf (39.7%) and 120 on artificial turf (60.3%). Of the 891 games played in this three-year period, 396 were played on natural turf (44.4%) and 495 were played on artificial turf (55.6%). Natural turf saw an injury rate of 0.199 per game, and artificial turf saw 0.242 injuries per game. We determined that there is no significant difference in rates of season-ending lower-extremity injury between artificial and natural turf. Any perceived difference in injury rates could possibly be attributed to the increased amount of play on artificial surfaces and anecdotal evidence related to high profile cases. While there is no significant difference in incidence, surfaces should continue to be monitored and regulated for traits such as hardness, and player preferences should be considered for qualities that are not quantifiable.

## 1. Introduction

A current controversial topic amongst National Football League (NFL) players, coaches, and organizations, is the risk of injury on artificial turf fields versus natural grass field surfaces. Data recently released by the NFL Players Association (NFLPA) stated there is a significantly higher incidence of injury on turf fields when compared with grass fields [1]. However, the NFL has made statements about these findings such as “The committee, including the NFLPA's experts, believe that simply playing on natural grass is not the answer to this complex challenge. Some artificial turf surfaces have a lower injury rate than some grass fields—and some grass fields have a lower injury rate than some artificial surfaces.” [2, 3]. Dallas Cowboys owner Jerry Jones stated, “Our league stats don't see issues with the type of surface that we have as opposed to natural grass,” and “We don't see issues. No facts bear that out.” [4]. A study conducted by Mack et al. concluded that NFL play on synthetic turf resulted in 16% more injuries per play than that on natural grass across all lower extremity injuries causing a player to miss any football participation [5]. Similarly, a study by Looughran et al. produced analogous results in NCAA football players, finding artificial turf is an important risk factor for specific knee ligament injuries in NCAA football. Injury rates for PCL tears were significantly increased during competitions played on artificial turf as compared with natural grass. Lower NCAA divisions (II and III) also showed higher rates of ACL injuries during competitions on artificial turf versus natural grass [6]. The association between artificial turf surfaces and ACL tears is demonstrated in literature [7, 8] as well as the incidence of lower extremity injury [9]. Previous studies have focused on the frequency of injuries occurring on artificial turf, or on ACL tears specifically; however, there is a gap in the literature when it comes to the incidence of season-ending injury in relation to grass and artificial turf fields. The purpose of this study is to examine the relationship between season-ending lower extremity injury and playing surface type.

## 2. Methodology

### 2.1. Study Design

Injury information on NFL players was obtained using publicly available data (https://fantasydata.com [8], https://profootballreference.com [10], https://spotrac.com [11], https://ESPN.com [12], https://NFL.com [13]) from the 2020, 2021, and 2022 seasons. Characteristics of the injury were researched and recorded as well as the specific game, date, stadium, and playing surface where the injury occurred. Data were entered into Excel and analyzed using two-sample *T*-tests.

### 2.2. Injury Classification

Lower extremity injury was classified by knee, lower leg, and ankle, and foot injuries. A season-ending injury was classified as an injury where, had it occurred in the first week, would typically render the individual unable to participate in any of the following games in the same season. The specific injuries included are generally considered to be season-ending regardless of the week in which they occurred. For our purposes, these categories include knee ligament tears (anterior cruciate ligament, posterior cruciate ligament, lateral collateral ligament, and medial collateral ligament), knee tendon tears (patellar tendon, quadricep tendon, and hamstring tendon), leg or ankle fractures, and Achilles tendon ruptures.

### 2.3. Inclusion/Exclusion Criteria

Only season-ending injuries which involved the knee, lower leg, or ankle were included. Injuries occurring in practice, off-season, and postseason (playoffs) were excluded. Only in-game injuries, including those occurring in preseason games, were included. Meniscus tears as well as sprains and strains to the aforementioned anatomical areas were excluded due to their wide range of severity and return to sport. Any injury that was undisclosed or could not be clearly identified was excluded.

### 2.4. Field Surface Classification

A surface with any proportion of artificial turf was included in the artificial turf category. This includes “hybrid” surfaces often comprised of mixtures 95% natural grass and 5% artificial turf, as the composition of the surface remains constant across the entire field and increases the durability of the entire playing surface uniformly. Only fields with 100% natural grass were included in the natural grass category.

## 3. Results

Over the course of the 2020, 2021, and 2022 seasons, there were a total of 199 season ending lower extremity injuries. 79 of those injuries occurred on natural turf surfaces, while 120 occurred on artificial turf surfaces. In those 3 years, 396 games were played on natural turf and 495 games were played on artificial turf. Accounting for number of games played on each surface, there was a rate of 0.199 injuries per game on natural turf (95% CI [0.155, 0.241]), and 0.242 injuries per game on artificial turf (95% CI [0.191, 0.275]). A breakdown of individual injury categories by year is shown in Table 1.

**Figure 1:**
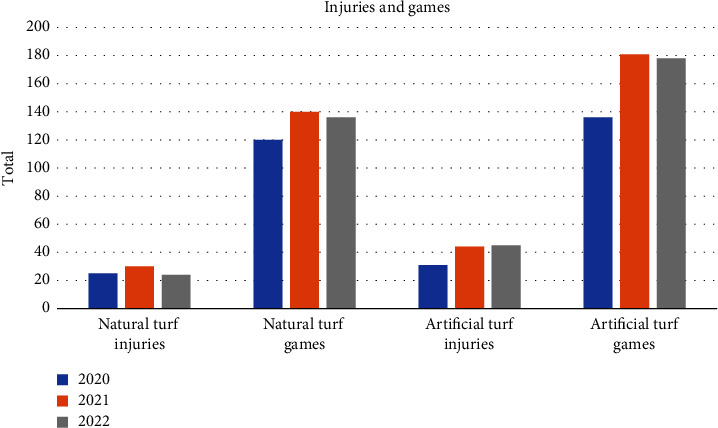
Visual demonstration of the number of season-ending injuries compared to the number of games on each surface.

In 2020, natural turf accounted for 25 qualifying season-ending injuries over the course of 120 games (0.208/game, 95% CI [0.126, 0.280]), while artificial turf accounted for 31 in 136 games (0.228/game, 95% CI [0.153, 0.289]). In 2021, natural turf saw 30 injuries in 140 games (0.214/game, 95% CI [0.116, 0.312) while artificial turf saw 44 in 181 games (0.243/game, 95% CI [0.131, 0.313]). Lastly, in 2022, natural turf saw 24 injuries in 136 games (0.176/game, 95% CI 0.097, 0.217]), and artificial turf saw 45 in 178 games (0.253/game, 95% CI [0.129, 0.357]). These findings are demonstrated in Figure 1.

Two-tailed *T*-tests comparing the injury rates of natural turf and artificial turf for each year and for the total 3-year period produced *p* values of 0.72 in 2020, 0.90 in 2021, 0.19 in 2022, and 0.25 for the entire 3-year period.

## 4. Discussion

There are many theories and a variety of anecdotal evidence as to whether one surface is more prone to injuries than the other. A significant amount of resources goes into developing and preparing the various artificial turf surfaces in order to make them as similar to natural grass as possible while enhancing the durability, drainage, and cost effectiveness of them. However, there is no way to truly predict how a surface will affect players in a game environment until it is installed for use. While it is shown here that artificial turf accounted for a greater total number of season-ending injuries, it also had a greater number of games played in all three seasons, as previously demonstrated in Figure 1.

Artificial turf surfaces have continued to grow in popularity with NFL franchises to the extent that more than half of teams currently use artificial turf at their home stadium [14]. This is reflected by the fact that, over the course of three years, artificial turf accounted for 495 (56%) games and 120 (60%) the injuries. When correcting for the amount of time played on each surface, injuries per game demonstrated no significant difference between the surfaces in any single season, as well as over the entire 3-year span.

Our study has shown that, while artificial turf does in fact see more total season-ending injuries, it is likely because artificial turf sees more play. However, there remains a large amount of anecdotal evidence against artificial turf. This can possibly be attributed to the players' general dislike for the surface. There have been a number of high-profile injuries on artificial turf that have spurred notoriety for the surface, however these cases comprise a very small sample size, and the data does not support this. While we have failed to demonstrate a significant difference between the two surfaces when it comes to season-ending lower extremity injuries, we must keep in mind that this study looked at a large cohort for several different injuries. There may be differences between surfaces for individual injuries themselves, such as the often-studied ACL tear or more common and less severe injuries. There may also be differences in the different subtypes of artificial turf, however small sample sizes for each would make this difficult to reliably determine. Lastly, there are many reasons to compare playing surfaces beyond just our select group of injuries here. Players often cite various features of surfaces such as hardness when hitting the ground and playability in the rain when discussing their preferences, all of which should be taken into consideration when selecting a playing surface.

There is limited evidence focusing on the topic discussed in this study in relation to NFL players. When expanding to compare our findings with other articles that covered multiple sports, the current body of evidence shows agreement with our results. Our results only begin to differ when compared to studies that subcategorized the injury types, which ours did not do [5, 7, 15]. One systematic review found that in many of the current articles evaluating lower extremity injury rates on artificial turf and natural grass, there was no significant difference in lower extremity injuries between the two playing surfaces. It is important to note that when this study restricted their data to “football being played at high-levels of competition,” players were more likely to suffer from knee injuries when playing on artificial turf when compared to natural grass [7].

It seems that once injuries are further subcategorized by lower extremity region (knee, ankle, etc.), results often show a significant difference between the two playing surfaces. One study that including injury data amongst NFL players from 2000 to 2009 agrees with this finding, demonstrating a significantly higher rate of knee and ankle sprains on artificial turf than natural grass [15]. Consequently, the results become mixed and more complex when looking at specific injury type and region. The aforementioned study offers an interesting point of comparison because it was performed using data from a time where there were more games played on natural grass than artificial turf. Although our study controls for differing amounts of play on each surface, this highlights how there has been a momentous change in the dominant playing surface in the NFL and compliments our investigation by looking at artificial turf injury rates when the number of games played on this surface was less prevalent.

Our study does not come without limitations. We were unable to classify noncontact vs contact injuries, which has been previously shown to have a significant difference, with increased incidence of non-contact injuries occurring on artificial turf [5]. Additionally, surfaces can be difficult to categorize due to the introduction of “hybrid surfaces.” For the purposes of our study, we considered any surface reinforced with any percentage of artificial fibers to be “artificial turf.” Our study only includes 3 years of player injury data and is exclusively publicly available data, which limits our ability to analyze individual injury types due to small sample sizes. Lastly, while *T*-tests best describe our data and our outcomes, they do not control for confounding variables, the possibility of which should always be taken into consideration when interpreting results. Season-ending lower extremity injuries comprise only a small number of injuries encountered in the NFL. No conclusions should be drawn from this data regarding incidences of other injuries in relation to playing surface or not.

Future directions of study should include updating the body of evidence for this topic for a wider range of years. This would allow for more data to be included, and possibly permit further subcategorization of injury (like mechanism of injury) to yield a more detailed analysis. Playing surface has become a more frequently visited topic in regard to the NFL and other major sports. Research in this field is also becoming increasingly more important due to the possibility of highlighting an addressable measure of player safety and injury prevention at all levels.

## 5. Conclusion

Our results show that, while season-ending lower extremity injuries are more common on artificial turf, the difference is not statistically significant when accounting for the increased amount of play seen by this surface. Variation occurs between all surfaces, even of the same type, artificial or natural. Surfaces should continue to be regulated and monitored for traits such as hardness, and player preferences should be considered for qualities that are not quantifiable.

## 6. Perspective

It is evident that while artificial turf has been discussed amongst NFL players, coaches, and the media with an increased risk of severe injury compared to natural grass, this discrepancy is largely attributable to the higher frequency of play on artificial surfaces rather than an inherent risk posed by the surface itself. This analysis, spanning three NFL seasons, challenges this narrative and underscores the complexity of attributing injury rates to playing surfaces. This finding is crucial for sports medicine; it suggests that current surface preferences and injury prevention strategies may need reevaluation. It also highlights the importance of considering play frequency in future research on sports-related injuries. The potential impact of these findings on sports medicine is significant, encouraging a broader and more nuanced approach to understanding the mechanisms of injury on turf and grass playing surfaces. This perspective may guide future policies and practices in sports field management, athlete preparation, and injury prevention strategies, ultimately enhancing player safety and performance.

## Figures and Tables

**Table 1:** Injury categories specific to each of the studied years.

2020	Artificial	Natural	Total
Knee ligament tears	24	14	38
Knee tendon tears	1	5	6
Achilles rupture	6	4	10
Ankle fracture	3	4	7
Lower leg fracture	1	0	1
Total	35	27	62

**2021**	**Artificial**	**Natural**	**Total**

Knee ligament tears	30	21	51
Knee tendon tears	4	2	6
Achilles rupture	8	1	9
Ankle fracture	2	3	5
Lower leg fracture	2	5	7
Total	46	32	78

**2022**	**Artificial**	**Natural**	**Total**

Knee ligament tears	30	12	42
Knee tendon tears	5	5	10
Achilles rupture	7	3	10
Ankle fracture	2	1	3
Lower leg fracture	4	2	6
Total	48	23	71

*Note:* Note that total injuries may be greater than number of injured players due to players experiencing concomitant injuries.

## Data Availability

Data for this study was collected from publicly available databases including https://fantasydata.com, pro https://footballreference.com, https://spotrac.com, https://ESPN.com, and https://NFL.com.
